# Complete Genome Sequence of *Pseudomonas* sp. Strain KUIN-1, a Model Strain for Studies on the Production of Cell-Free Ice Nucleation Proteins

**DOI:** 10.1128/MRA.01204-19

**Published:** 2019-11-07

**Authors:** Taisei Yamamoto, Yoshie Hasegawa, Hidehisa Kawahara, Hiroaki Iwaki

**Affiliations:** a Department of Life Science and Biotechnology, Kansai University, Suita, Osaka, Japan; Georgia Institute of Technology

## Abstract

*Pseudomonas* sp. (formerly Pseudomonas fluorescens) strain KUIN-1 is an ice-nucleating bacterium that was isolated from the leaves of field beans (Phaseolus vulgaris L.). This microorganism can release cell-free ice nucleation proteins and shows cold shock-induced freezing tolerance. Here, we report the 6,028,589-bp complete genome sequence of *Pseudomonas* sp. KUIN-1.

## ANNOUNCEMENT

Pseudomonas sp. (formerly Pseudomonas fluorescens) strain KUIN-1 is an ice-nucleating bacterium that was isolated from the leaves of field beans (Phaseolus vulgaris L.), grown on Ellerslie Farm at the University of Alberta, Canada, by Obata et al. (1). Bacterial ice nuclei (or ice nucleation proteins) are outer membrane-associated lipoglycoproteins that are widely used in artificial snow production and also have the potential to be utilized as cloud-seeding agents and in the processed and frozen food industries (2). However, their commercial applications have been hampered because they are normally produced by plant-pathogenic species, such as those from the genera Pseudomonas and Pantoea (synonym Erwinia) (2). *Pseudomonas* sp. strain KUIN-1 releases cell-free ice nucleation proteins (3), and the cell-free character of the proteins makes it unnecessary to consider its plant-pathogenic character when utilizing them. Additionally, strain KUIN-1 shows cold shock-induced freezing tolerance (4). Here, we report the complete genome sequence of *Pseudomonas* sp. KUIN-1 in order to facilitate its further characterization and taxonomic identification.

*Pseudomonas* sp. strain KUIN-1 was provided to us by Hitoshi Obata (Kansai University, Osaka, Japan), and the genomic DNA was isolated from cells after culture for 18 h in 50 ml of Miller’s LB medium (Merck Millipore) at 30°C, using Wilson’s procedure with some modifications (5). Cells were then washed twice with Tris-EDTA buffer and then resuspended in 15 ml of the same buffer supplemented with 1 mg/ml lysozyme; the amounts of the subsequent reagents were scaled up in relation to the volume of the cell suspension. A 20-kb SMRTbell template library was prepared from approximately 8 μg of input genomic DNA, using the SMRTbell template prep kit 1.0 (Pacific Biosciences). The SMRTbell library was sequenced using single-molecule real-time (SMRT) cell 8Pac version 3 and P6-C4 chemistry, and 240-min movies were captured for each SMRT cell using the PacBio RS II instrument (Pacific Biosciences). The default parameters were used for all software, unless otherwise noted. Subreads were filtered using PreAssembler Filter version 1 (minimum subread and polymerase read lengths. 500 and 100 bp, respectively; minimum polymerase read quality, 0.80) in SMRT Analysis version 2.3.0 (Pacific Biosciences), and 59,461 reads, composed of 609,693,648 bp, with an *N*_50_ value of 16,730 bp, were obtained. The reads were *de novo* assembled with the Hierarchical Genome Assembly Process (HGAP) protocol version 3 in SMRT Analysis to produce one circular contig with 85× coverage depth (6). The complete genome sequence of strain KUIN-1 consisted of a 6,028,589-bp circular chromosome that had a G+C content of 59.23%, and no plasmid was found.

Strain KUIN-1 was reclassified using the 16S rRNA gene sequence, digital DNA-DNA hybridization (dDDH), and average nucleotide identity (ANI) (Table 1). The 16S rRNA gene sequence identities were calculated using the BLASTN program (https://www.ncbi.nlm.nih.gov/BLAST/) from the National Center for Biotechnology Information against the 16S ribosomal RNA sequences (*Bacteria* and *Archaea*) database. The ANI values were calculated with JSpeciesWS (http://jspecies.ribohost.com/jspeciesws/) using BLAST (7, 8). The dDDH values were calculated with the Genome-to-Genome Distance Calculator 2.1, available on the DSMZ website (https://ggdc.dsmz.de), using formula 2 (9, 10). The 16S rRNA gene sequence analysis revealed that strain KUIN-1 is affiliated with the Pseudomonas syringae group (11) rather than with Pseudomonas fluorescens (Table 1). The ANI and dDDH values were determined by using the five species that were most closely related to strain KUIN-1 based on the 16S rRNA gene sequence analysis. The ANI and dDDH values showed that strain KUIN-1 was most closely related to P. syringae (Table 1), and that these values were slightly below the accepted threshold for prokaryotic species boundaries, which are 95 to 96% for ANI and 70% for dDDH. This indicates that although strain KUIN-1 is closely related to P. syringae, the strain should be classified as a novel species in the genus *Pseudomonas*. Therefore, we tentatively identified strain KUIN-1 as a *Pseudomonas* sp.

**TABLE 1:** 16S rRNA gene sequence identity and ANI values for *Pseudomonas* sp. strain KUIN-1 compared to closely related species

*Pseudomonas* species	16S rRNA gene % identity (GenBank accession no.)	dDDH^*a*^ (%)	ANI^*b*^ (%)	Genome sequence GenBank accession no.
P. congelans	99.87 (NR_028985)	55.8	93.61	FNJH00000000
P. cerasi	99.86 (NR_146827)	57.4	93.74	LT222319
P. syringae	99.73 (NR_043716)	60.6	94.65	JALK00000000
P. savastanoi	99.72 (NR_117822)	37.1	88.26	LJRJ00000000
P. cannabina	99.60 (NR_025550)	30.5	84.84	FNKU00000000
P. fluorescens	97.96 (NR_115715)	22.6	76.33	LT907842

a dDDH, digital DNA-DNA hybridization.

b ANI, average nucleotide identity.

The genome sequence was annotated with the DNA Data Bank of Japan (DDBJ) Fast Annotation and Submission Tool (DFAST; https://dfast.nig.ac.jp) (12). In total, 5,230 protein-coding sequences, 63 tRNA genes, and 16 rRNA genes were detected. As expected, because this species produces ice nuclei (3), one gene encoding the ice nucleation protein (1,352 amino acids) was predicted in the genome of strain KUIN-1. The genome also contains five CspA cold shock protein-like genes (13), which are considered to function as RNA chaperones and to prevent the formation of the secondary structures of mRNAs that enable efficient translation of mRNAs at low temperatures (14). The complete genome sequence will help clarify the mechanisms involved in the release of the ice nucleation proteins and the cold shock-induced freezing tolerance of strain KUIN-1.

### Data availability.

The genome sequence reported here was deposited in DDBJ under accession number AP020337. The associated BioProject, BioSample, and DDBJ Sequence Read Archive (DRA) accession numbers are PRJDB8624, SAMD00182269, and DRA009026, respectively.
